# Strategy Choice Mediates the Link between Auditory Processing and Spelling

**DOI:** 10.1371/journal.pone.0107131

**Published:** 2014-09-08

**Authors:** Tru E. Kwong, Kyle J. Brachman

**Affiliations:** Department of Psychology, Mount Royal University, Calgary, Alberta, Canada; MRC Institute of Hearing Research, United Kingdom

## Abstract

Relations among linguistic auditory processing, nonlinguistic auditory processing, spelling ability, and spelling strategy choice were examined. Sixty-three undergraduate students completed measures of auditory processing (one involving distinguishing similar tones, one involving distinguishing similar phonemes, and one involving selecting appropriate spellings for individual phonemes). Participants also completed a modified version of a standardized spelling test, and a secondary spelling test with retrospective strategy reports. Once testing was completed, participants were divided into phonological versus nonphonological spellers on the basis of the number of words they spelled using phonological strategies only. Results indicated a) moderate to strong positive correlations among the different auditory processing tasks in terms of reaction time, but not accuracy levels, and b) weak to moderate positive correlations between measures of linguistic auditory processing (phoneme distinction and phoneme spelling choice in the presence of foils) and spelling ability for phonological spellers, but not for nonphonological spellers. These results suggest a possible explanation for past contradictory research on auditory processing and spelling, which has been divided in terms of whether or not disabled spellers seemed to have poorer auditory processing than did typically developing spellers, and suggest implications for teaching spelling to children with good versus poor auditory processing abilities.

## Introduction

Does what people hear affect how they spell? Certainly in an extreme case, such as when one mistakes one word for another (e.g., thinking one has heard the word *train* when the speaker has actually said *chain*), accurate spelling is unlikely. What, however, of less extreme cases? For instance, what should one expect from a person who does not distinguish between two different phonemes (speech sounds) as accurately as does another person? Will this inability to make a phonemic distinction affect an individual's ability to spell words containing those phonemes? Will it affect the individual's ability to spell in general?

Moreover, if linguistic auditory processing *does* affect spelling ability, the question remains as to why. Are such problems specific to language, or does this difficulty have roots in more basic level sounds? If one person is better at discriminating among auditory frequencies than is another, does this discriminative ability provide information about these individuals' respective spelling abilities? The ability to distinguish between two tones may seem quite removed from the ability to perceive and spell phonemes. However, could this basic auditory ability (or disability) be representative of some lower level of processing? In the case where basic auditory processing is compromised, this issue may be considered a possible underlying cause for linguistic auditory processing difficulties and may result in spelling difficulties. The goals of the current research were threefold: to determine whether or not there is a basic link between nonlinguistic and linguistic auditory processing, to look for links between these types of processing and spelling ability, and to determine whether or not spelling strategy choice mediates those links.

Much of the research in this area has been conducted with children learning to spell and with children and adults experiencing reading difficulties. The present study extends these lines of research to examine the role of auditory processing in spelling for nondisabled adults.

### Linguistic Auditory Processing

The literature concerning the effects of linguistic auditory processing on spelling is largely clear and consistent, at least with regard to learning to spell in English. Researchers [54] compared speakers of different English dialects and found that differing pronunciations of certain words led to differing spellings among beginning spellers. Research [51] has found that the effect of pronunciation on spelling may persist into adulthood. She compared a group of college students who spoke African American vernacular English (AAVE) to a group of Caucasian students, and found that differences in pronunciation of the final/d/(often pronounced more as/t/in AAVE) influenced spelling in these adult participants.

The effects of pronunciation on spelling in other contexts have also been examined. For instance, researchers have studied children's spellings of syllabic consonants; that is, cases in which the rime of a syllable is similar or identical to the letter-name of the final consonant [40], [52]. In these studies, it was found that beginning spellers often omitted the preceding vowel in cases like this (e.g., spelling *car* without the *a*), presumably because the presence of the vowel is not clear to them in the pronunciation of the words.

Such research shows that there are conditions that can be present in dialect or in a word's structure that can cause letters to be inserted or omitted in spelling and pronunciation. How common is it, though, for two phonemes to be confused? In cases where the pronunciation of two phonemes differs only in one characteristic, such as voicing (whether or not the vocal cords vibrate during articulation), young children often have difficulty distinguishing between the two [53]. Is it reasonable to assume, then, that these confusions affect their spelling? Other researchers [55] found that children who had difficulty discriminating between confusable phonemes had lower spelling accuracy, not only when attempting to spell words that contained those phonemes, but also for other words.

It has been found that phonological awareness, measured in preschool children, predicted reading and spelling in Grade 1 in Both English and Dutch children [19]. A study with English-speaking Grade 1 children, showed a relationship between phonemic segmentation ability and spelling [16]. It has also been found that early phonological awareness (tested in preschoolers) correlated with spelling skills at school age [33].

When considering research focused on reading, much of it focusing on reading disabilities, we see perhaps an even clearer influence of auditory processing than when looking at spelling. For instance, it has been repeatedly demonstrated different ERP responses to presentation of phonemes for infants who are at familial risk for dyslexia and those who are not [21], [22], [30]–[32], [39]. This indicates differences in the brain's processing of phonemes before an individual even begins learning to read and write.

A longitudinal study of children with familial risk for dyslexia yielded evidence of a relationship between linguistic auditory processing and literacy skills. The 5-year-olds included in the initial study were more likely to show deficits in speech-in-noise perception if they were from families with risk of dyslexia [6]. The children's phonological awareness was later tested in terms of their ability to parse out the different sounds in words [8]. Their performance on these tasks not only correlated with their literacy skills at that point in time, but predicted literacy skills in Grade 1. In another follow-up with the same participants, the researchers found that impaired phonological awareness in Kindergarten predicted the diagnosis of dyslexia by Grade 3 [7].

Researchers working with English-speaking adults have found deficits on almost all measures of speech perception among reading disabled participants [57]. In one study, children with reading disabilities performed more poorly on phoneme discrimination tasks than did those with no disabilities [10]. In another, children who were poor (but not necessarily diagnosed as disabled) readers, showed deficits in phoneme discrimination as well [15]. It has also been shown that children who are poor readers have more difficulty in phoneme discrimination than do good readers [37]. In fact, one study indicated that reading problems could be predicted with 92% accuracy using a combination of several measures of phonological awareness [20].

Taken together, the presented research suggests a general agreement that baseline linguistic auditory processing is lower in individuals diagnosed with disabilities in reading or spelling. In the case of nonlinguistic auditory processing, however, there is considerably less agreement.

### Nonlinguistic Auditory Processing

There is considerably less agreement concerning the effects of nonlinguistic auditory processing on spelling than there is concerning linguistic auditory processing. Researchers' lack of agreement concerning the effects of nonlinguistic auditory processing on spelling ability is not due to a lack of research in the field. Over 30 years ago, Tallal and her colleagues were investigating possible links between nonlinguistic auditory processing and reading and writing skills

#### Studies finding an effect

Tallal found that primary school children with reading disabilities performed as well as normal readers when the tones were presented relatively slowly, but not when tones were presented rapidly [48]. Working with colleagues, she found that performance on auditory tasks could be used to classify primary school children as having or not having language impairments with near-perfect accuracy [50]. Children who had language impairments almost always performed significantly more poorly on the task than did those who did not.

Since then, researchers have found differences between individuals with and without reading or language impairment in terms of ability to detect frequency changes in a sound [7]–[8], ERP (event-related potential) responses to tones differing in rise times [25], performance on tasks involving rise time and temporal order of sounds [41], frequency discrimination [24], [27], [36].

In a study with university students, adults with good auditory temporal processing ability (as determined by tasks such as gap detection and determining the order in which two tones have been presented) were better readers and spellers than those with poor auditory temporal processing [3].

Both of the auditory impairments (i.e., discrimination and sequencing) that Tallal and her colleagues reported in children who had reading disabilities and language impairments have been replicated. One group of researchers found that adults with dyslexia were less able to discriminate between tones of differing frequencies; they required larger differences between two tones, on average, in order to detect a difference [34], [46]. Others found that adults with dyslexia had more trouble with pitch discrimination tasks when the interstimulus interval (ISI) was brief [17]. Another group found that 7- to 13-year-old children who had language impairments were less accurate in their recollection of tone sequences than were unimpaired controls [5]. Several other research groups have found similar impairments when, as in [17], the ISI is brief, e.g., [9], [11]. However, a closer look at Stein and McAnally's [34], [46] results reveals that there were some mixed results in terms of which tasks elicited differential performance. Specifically, they did not find significant differences between readers with dyslexia and normal readers in the ability to detect very brief gaps between presentations of a tone, contrary to another of their predictions. Also, as discussed below, other researchers have reported evidence that contradicts their findings.

#### Studies finding no effect

Attempts have been made to replicate McAnally and Stein's [34] findings [28]. These researchers found that, once outliers were removed, most significant differences between participants with and without dyslexia disappeared; with the exception of a subgroup of participants with dyslexia, all of the differences disappeared. They concluded that any phonological deficit that may exist in dyslexia could not be reasonably connected to a low-level auditory deficit based on current evidence [28]. Some other researchers [10], [37] found no evidence of impaired frequency discrimination or temporal order judgment of non-speech sounds in reading disabled children compared to controls.

Some researchers have found no difference between the gap-detection threshold for groups with spelling disabilities and for control groups [44]. Others have found that children with dyslexia performed significantly worse than did the control group children on speech segmentation, but not on the non-speech tasks [38].

Some of the contradictory results reported regarding the effects of nonlinguistic auditory processing on reading and spelling may be due to different measures of nonlinguistic auditory processing. Nonlinguistic auditory processing has been operationalized in terms of frequency discrimination, temporal order judgment, gap detection, and rise time discrimination, to name just a few. However, there has not been complete agreement of results even when similar measures have been used. Some researchers [28], for instance, were unable to completely replicate McAnally and Stein's [34] results. Their discovery of a subgroup of individuals with dyslexia for which there was a significant correlation, though, does suggest that the conflicting results could be explained, at least partially, by differences in participant groups. Specifically, participants who have less accurate nonlinguistic auditory processing may be compensating for this impairment to different degrees. Another possibility is that different ISI lengths contributed to different results, and that temporal issues (determining order of presentation, or length of gap between tones), rather than simple discrimination (whether or not two tones are of the same frequency) are responsible for some of the results.

### Spelling Strategy as a Possible Mediating Variable

One possible reason why, some researchers find a correlation between auditory processing and spelling ability while others do not may be that some individuals with less accurate auditory processing are compensating for processing deficits. One form of compensation is strategy choice. This idea is supported by findings obtained when researchers had children with and without dyslexia (matched for reading level) read information from a computer screen at different speeds and in both a quiet condition and a condition in which auditory masking (that is, white noise presented via headphones) was used [12]. Both auditory masking and reading acceleration can function to shift the reader's focus away from linguistic auditory processing and towards other strategies. These conditions combined served to enhance the reading performance of children with dyslexia only. It seems plausible that these children may have had less accurate nonlinguistic and/or linguistic auditory processing and thus may have performed better when using strategies other than phonology. For individuals with more accurate auditory processing, phonology may be a more viable choice, so shifting attention away from phonological strategies would thus not be helpful. Given the connection between reading and spelling, it also seems plausible that this phenomenon may generalize to spelling ability. Also, McAnally and Stein [35] found their hypothesized differences between individuals with and without dyslexia in terms of amplitude modulation following response (AMFR; that is, a change in the potential recorded at the scalp following response to a tone stimulus) in only one of three frequency ranges when the two groups of participants were matched for hearing sensitivity. Thus, it is reasonable to ask if something other than auditory acuity is also influencing the difference in their reading and spelling abilities. The possibility exists that it stems from differences in the strategies the participants were using to perform these tasks. Specifically, we might expect to find differences based on whether or not individuals are relying heavily on phonological, or “sounding out” strategies.

The extent to which individuals who choose adaptively among spelling strategies (e.g., avoiding phonological strategies if auditory processing is less accurate) versus those who do not are included in a sample may have considerable implications for the results of that study. Research on strategy choice, e.g., [45] suggests that people are more likely to employ strategies that have been successful in the past, so we may expect that many individuals with less-accurate auditory processing will use strategies that are not based on phonology. However, that is not likely the case for all. If more of the participants in a study who have less-accurate auditory processing are also less-adaptive spellers, any link between auditory processing and spelling is likely to be clear. People who fail to “sound out” words accurately, but continue to try, are likely to show deficits in spelling. If more of the participants in a study who have less-accurate auditory processing are adaptive spellers, they presumably do not “sound out” many words. Thus, they are less likely to show a deficit in spelling, as they have found ways to compensate for this potential source of spelling difficulty. In this case, even if there is a link, or potential link, between auditory processing and spelling accuracy, it would not be evident by testing these participants.

### Research Needs

While there is a plethora of literature on the subject of auditory processing and its connection to reading and spelling accuracy, there are several conspicuous holes in the research. Particularly unclear is the relationship between linguistic and nonlinguistic processing. That is, it is unclear whether or not individuals with less accurate nonlinguistic auditory processing are also likely to have less accurate linguistic auditory processing.

Also, nonlinguistic auditory processing, as it relates to literacy skills, has been studied almost exclusively in individuals who have reading and/or language impairments, e.g., [17], [23] [46], [49]. In an exception, one group of researchers found that children with better nonlinguistic auditory processing (as demonstrated in a frequency discrimination task) did show better literacy skills [47]. However, even this study may have been measuring a distinction between participants with and without impairments; the effect related specifically to readers in the lower quartile of their participant group, and they did not exclude participants on the basis of learning disabilities. The literature on linguistic auditory processing has been expanded to include individual differences within typically-developing populations. It has been shown that linguistic auditory processing skills affect spelling even within normal readers and spellers [55]. Light may be shed on the area of nonlinguistic auditory processing if such differences are investigated.

In addition, other variables may mediate the effect of auditory processing on spelling. It is possible that there is a mediating variable, such as spelling strategy use, influencing the link between auditory processing and spelling ability. As described above, an argument can be made for the viability of strategy choice as a means through which a person may circumvent auditory processing difficulties. It is also possible that there are other mediating variables that have not been explored.

We are left with indications that the different types of auditory processing are quite possibly linked to one another, clear indications of links between linguistic auditory processing and spelling, and less clear indications of links between nonlinguistic auditory processing and spelling. There is reason to believe spelling strategy may mediate these links, but little to no direct assessment of this likelihood. There has also been very little research exploring whether or not auditory processing influences individual differences in spelling for typically-developing individuals. Thus, the specific questions addressed in the present study are: a) whether or not the different types of auditory processing even correlate with one another, b) which (if any) of the types of auditory processing correlate with spelling ability in a group of individuals without reading disabilities, and c) whether or not strategy choice mediates any relationship between auditory processing and spelling ability.

### Rationale for Measures

Both auditory processing tasks were chosen largely for their simplicity. Although some very important work is being done to determine the exact nature and origin of any auditory processing deficits, at least among individuals with dyslexia, e.g., [1]–[2], [56], the purpose for the present study is best served by using the simplest measures possible. Our interest in nonlinguistic auditory processing here was in tone discrimination, rather than temporal ability. That is to say that we are interested in this initial exploration in participants' ability to distinguish tones of different frequencies, rather than in their ability to detect small gaps between tones or the temporal order of tones. In many studies of nonlinguistic auditory processing, ISI has not been reported. When it has, e.g., [17], the studies have involved brief ISIs for study of temporal processing. This does not necessarily allow temporal processing effects (e.g., is there a gap between the tones?) to be teased apart from discrimination (are the tones the same or different?). For this reason, our measure (described below) involves two-tone stimuli with a relatively long (0.5sec) ISI. A significant correlation between nonlinguistic auditory processing and spelling ability should clearly indicate an effect that is specific to frequency perception. A lack of correlation may suggest that past findings have been based on temporal processing, rather than problems with frequency perception.

Our test of nonlinguistic auditory processing was based on the tone discrimination task used by McAnally and Stein [34], but unlike that study, we presented all tones to all participants, regardless of performance. McAnally and Stein used the method of limits, starting with larger frequency differences, decreasing the difference when participants answered correctly. However, in an initial pilot trial of this task, we noticed that participants sometimes erred on a judgment for a larger frequency difference, only to perform much better on subsequent trials with smaller differences. Rather than indicating a participant's just noticeable difference, our method indicates a total number of correct response, allowing participants the opportunity to give correct answers on more difficult pairs even if they have given incorrect answers on simpler pairs.

One measure of linguistic auditory processing was phoneme perception, conducted in much the same way as was done by Varnhagen et al. [55]. Participants' scores were based on their percentage of correctly identified phonemes, to mirror the process used for nonlinguistic auditory processing. A second linguistic auditory processing task was a mirror of the “same/different” paradigm used in the nonlinguistic task. Because the phonemes were recorded for natural sound, rather than computer-generated, this task carries the disadvantage of the possibility of factors *other than* linguistic auditory processing (e.g., differences in white noise or tone of voice from one recording to the next) affecting performance. However, this task is arguably easier to compare to the “same/different” frequency discrimination task than is the letter-choice task.

We obtained strategy reports, while participants completed the spelling subtest of the *Wide Range Achievement Test 3 (WRAT 3)* [58], to determine participants' preferred strategies. Past research, e.g., [29], [42], [43] supports the use of self-reports of strategy use. We expected that participants' preferences for phonological or nonphonological strategies would affect the degree to which their nonlinguistic and linguistic auditory processing skills influenced their spelling ability. Assignment to a preferred-strategy category was based on a median split. Previous research [29] demonstrated that most individuals have some clear preference in terms of spelling strategy.

We measured participants' spelling ability using words taken from the vocabulary tests that are included in the *Test of Written Language – 4^th^ Edition (TOWL 4)* [26]. Because the tasks were being completed by a university student population, it was necessary to make the task challenging; many of the vocabulary words from the TOWL 4 were more difficult than were those from the standardized spelling test, and were thus compiled into a test. As participants were all adults with comparable levels of education, unstandardized scores should still yield usable results.

## Method

### Ethics Statement

Ethics approval was obtained from Mount Royal University's Human Research Ethics Board, application number 2011–38. Written consent was obtained from all participants.

### Participants

Participants were 63 undergraduate students (50 female, 13 male) enrolled in introductory psychology courses in an undergraduate university in Alberta, Canada. Participants received course credit for participation. Criteria for participation included requirements that participants have English as their first language and not be diagnosed with any type of disability affecting literacy. An additional five female participants were excluded from analysis because they did not meet the criteria for the study or because they spoke accented English that could have potentially influenced their phoneme perception. Ages were not collected, as none of the tests used were standardized.

### Materials

#### Nonlinguistic auditory processing

Tones for the nonlinguistic auditory processing task were presented via headphones on computer. There were three groups of tones: one in the range of 1000 Hz, one in the range of 1500 Hz, and one in the range of 2000 Hz. For each pair of tones, one tone was exactly 1000, 1500, or 2000 Hz. For two presentations within the set, the tones would match (two ones at 1000 Hz, for example). Within each range, there were two presentations in which the tones differed by 2 Hz, two in which the tones differed by 4 Hz, with the difference increasing by 2 Hz intervals up to a maximum of 20 Hz. At each difference level, one of these tones was lower than the baseline and one was higher (e.g., one set pairing 1000 Hz with 1002 Hz, and one set pairing 1000 Hz with 998 Hz). This resulted in a total of 66 tone pairs presented to each participant (Appendix S1 lists all tone pairs); the task was scored in terms of total number of pairs correctly identified as same or different, and also in terms of median reaction time (RT) for items. The order in which the tone pairs were presented was randomized for each participant. Three practice pairs (either identical or very different) were presented as well, to ensure all participants understood instructions.

#### Linguistic auditory processing

Phonemes were presented via headphones on computer. For the phoneme discrimination task, participants heard pairs of phonemes. The phonemes were sometimes identical, sometimes similar, and sometimes dissimilar. For similar phonemes, or confusable phonemes, the phonemes always differed in a single feature of articulation. For most of these pairs, one phoneme was voiced and one was unvoiced. There were several pairs for which the difference was between fricative and lateral fricative or between dental and labiodental. For each pair, participants were required to indicate whether they were hearing two different phonemes (‘different’) or the same phoneme twice (‘same’). Each participant heard a total of 43 pairs of phonemes (see Appendix S2 for a complete list of phonemes pairs); 41 of these pairs were heard twice, while 2 pairs that were part of multiple confusable phoneme sets (b/b, which was part of the b-d and b-p pairs; and f/f, which was part of the f-v and f-θ pairs) were heard four times, for a total of 92 trials. The task was scored in terms of total number of pairs correctly identified as either same or different, and also in terms of median RT for responses.

For the phoneme spelling task, participants heard single phonemes. For each, they were asked to choose among three potential spellings: one correct, one spelling for a similar phoneme, and one spelling for a dissimilar phoneme. The confusable phoneme pairs were the same as for the “same/different” task. There were 20 sets of phonemes and spellings (see Appendix S3 for a complete list of phonemes and response options); each was presented twice, for a total of 40 trials. The task was scored in terms of total number of correct responses, and also in terms of median RT for responses.

Practice items, presented to ensure that all participants understood instructions, consisted of pairs of words or sounds that either matched or were dissimilar.

#### Strategy choice

Words selected from the WRAT 3 [58] were used to obtain strategy reports. Fifteen words, selected to represent words whose spellings probably were and probably were not in participants' long-term memories (i.e., words that are quite common or very uncommon), were read aloud by one of the researchers. Strategy reports were ultimately scored as being phonological (based entirely or mostly on “sounding out” the word) or nonphonological (based mostly on strategies that do not involve “sounding out” the word, such as using orthographic conventions).

#### Spelling ability

Thirty-three words and sample sentences taken from the vocabulary tests of the *TOWL-4* [26] were read aloud to participants for the test of spelling ability. Only raw scores were used for this test.

### Procedure

Participants completed all tasks in individual sessions that took approximately 35 minutes. No feedback was provided on any task except for the practice trials that were presented at the beginning of each auditory processing task. Participants began with the nonlinguistic auditory processing task. They wore headphones to ensure standardized noise and volume conditions. Tones pairs were randomized throughout. Participants were to determine, for each pair, whether or not the tones matched; they responded to each tone pair by clicking either “same” or “different” on the computer screen.

Following completion of the nonlinguistic auditory processing task, participants began the phoneme discrimination task. Again, they wore headphones to ensure standardized presentation. Phoneme pairs were presented in random order. Participants again responded to each pair by clicking either “same” or “different” on the computer screen.

Third, participants completed the phoneme spelling task. Phonemes were presented in random order. For each phoneme, participants saw three spelling choices, also presented in random order. Participants used the mouse to select the spelling that they thought represents the presented phoneme.

The fourth task was the strategy choice task. One of the researchers read participants a word, a sentence containing the word, and the word again. Participants wrote the word using pencil and paper. After spelling each word, participants were asked to describe their strategies (e.g., “How did you decide how that word should be spelled?”). Non-leading probing was used for participants who did not give full explanations of spelling (e.g., “Is there anything else?”, “That explains how you spelled the beginning of the word. Can you tell me how you figured out how to spell the end?”).

The spelling ability tasks were presented last. For each word, the experimenter read a word, a sentence containing the word, and then the word again. Participants were given time to complete each spelling before moving on to the next word.

### Statistical Analyses

Data were analyzed in two ways: using correlations and a regression analysis. Pearson's correlations were conducted to investigate relationships among the three measures of auditory processing, and to investigate the relationship between each measure of auditory processing and spelling ability for each strategy grouping. A regression was used to investigate the potential influence of each auditory processing measure and of strategy choice on spelling ability.

## Results

### Strategy Scoring

Strategy reports, collected orally and retrospectively for each individual word, were assigned to one of several categories (see Table 1). Although responses were categorized by specific strategy or combination of strategies, the only categorization that is meaningful for our purposes is whether or not participants used *phonology*, sounding out a word. Strategy reports for any given word were designated as follows: “phonology”, ”half phonology” (if a participant sounded out part of a word, but spelled part using another strategy), or “not phonology”. Inter-rater reliability was checked by having 10% of the strategy reports scored independently by both raters. Raters agreed fully on 96% of the reports, partially agreed (e.g., agreeing on one strategy a participant described for a particular word, but disagreeing on another) on 2.8% of the others. Agreement was 100% for the extent to which any given word was spelled phonologically. For the purposes of further testing, a median split was used to divide participants into phonological (6 or more of 15 words spelled using phonology only) versus nonphonological (5 or fewer) spellers.

**Table 1:** Means and Standard Deviations for Accuracy in Auditory Processing and Spelling Tasks.

	Phonological Spellers		Nonphonological Spellers		Combined	
	*M*	*SD*	*M*	*SD*	*M*	*SD*
Nonlinguistic	29.00	13.02	29.06	11.02	29.03	11.96
Phon. Discrimination	86.53	7.44	86.34	11.29	86.44	9.55
Phoneme Spelling	39.4	0.93	39.81	0.40	39.61	0.74
Spelling Ability	24.77	4.57	27.09	3.23	25.95	4.09

### Descriptive Statistics

Scores on auditory processing measures are given in terms of number of correct responses. For nonlinguistic auditory processing, the maximum possible score was 66. For the phoneme discrimination task, the maximum possible score was 92. For the phoneme spelling task, the maximum possible score was 40. Reaction times were also recorded, and are presented as average time (in seconds) per item. There were four cases in which one of the tasks failed to record results (one nonlinguistic auditory processing, one phoneme discrimination, and two phoneme spelling); the participants in these cases were dropped from analyses including these tasks, but still included in the remainder of analyses. Spelling scores are also given in terms of total number correct, with a maximum possible score of 33. Table 1 shows the means and standard deviations for accuracy on the auditory processing tasks and the spelling ability task. Table 2 shows means and standard deviations for reaction times on the auditory processing tasks. Nonphonological spellers performed significantly better on the spelling test than did phonological spellers (*t*(61)  = 2.33, *p* =  .023), and slightly but significantly better on the phoneme spelling task (*t*(39)  = 2.20, *p* =  .034), but the two groups were otherwise comparable. Both groups showed ceiling effects for both linguistic auditory processing tasks (phoneme discrimination and phoneme spelling). These tasks are thus analyzed in terms of Reaction Time (RT) rather than accuracy.

**Table 2:** Means and Standard Deviations for Reaction Time (in seconds) in Auditory Processing Tasks.

	Phonological Spellers		Nonphonological Spellers		Combined	
	*M*	*SD*	*M*	*SD*	*M*	*SD*
Nonlinguistic	4.43	0.66	4.75	1.09	4.58	0.91
Phon. Discrimination	4.71	0.66	4.79	0.58	4.75	0.62
Phoneme Spelling	3.10	0.39	3.08	0.42	3.09	0.40

### Linguistic Versus Nonlinguistic Auditory Processing

**Figure 1:**
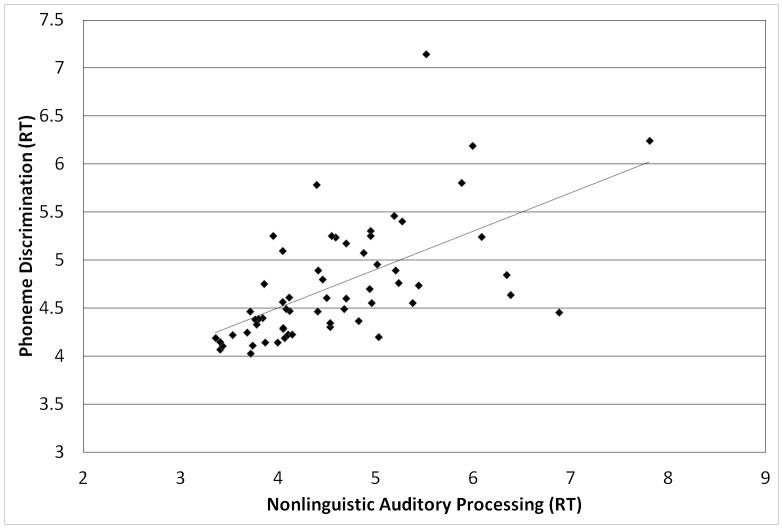
Correlations between RTs on nonlinguistic auditory processing and phoneme discrimination, *r*(59) =  .60, *p* < .001.

**Figure 2:**
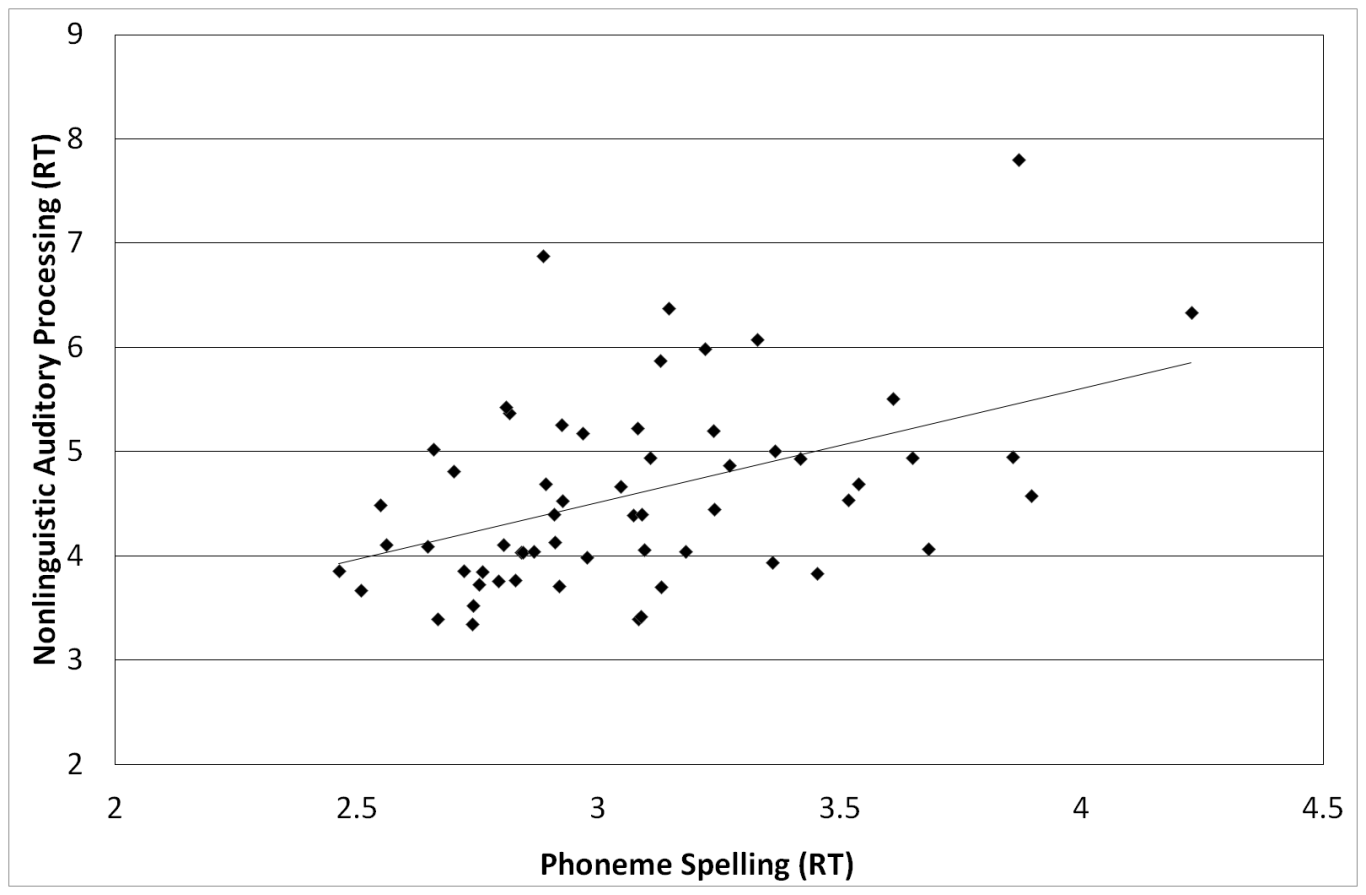
Correlation between RTs on nonlinguistic auditory processing and phoneme spelling, *r*(58) =  .45, *p* < .001.

**Figure 3:**
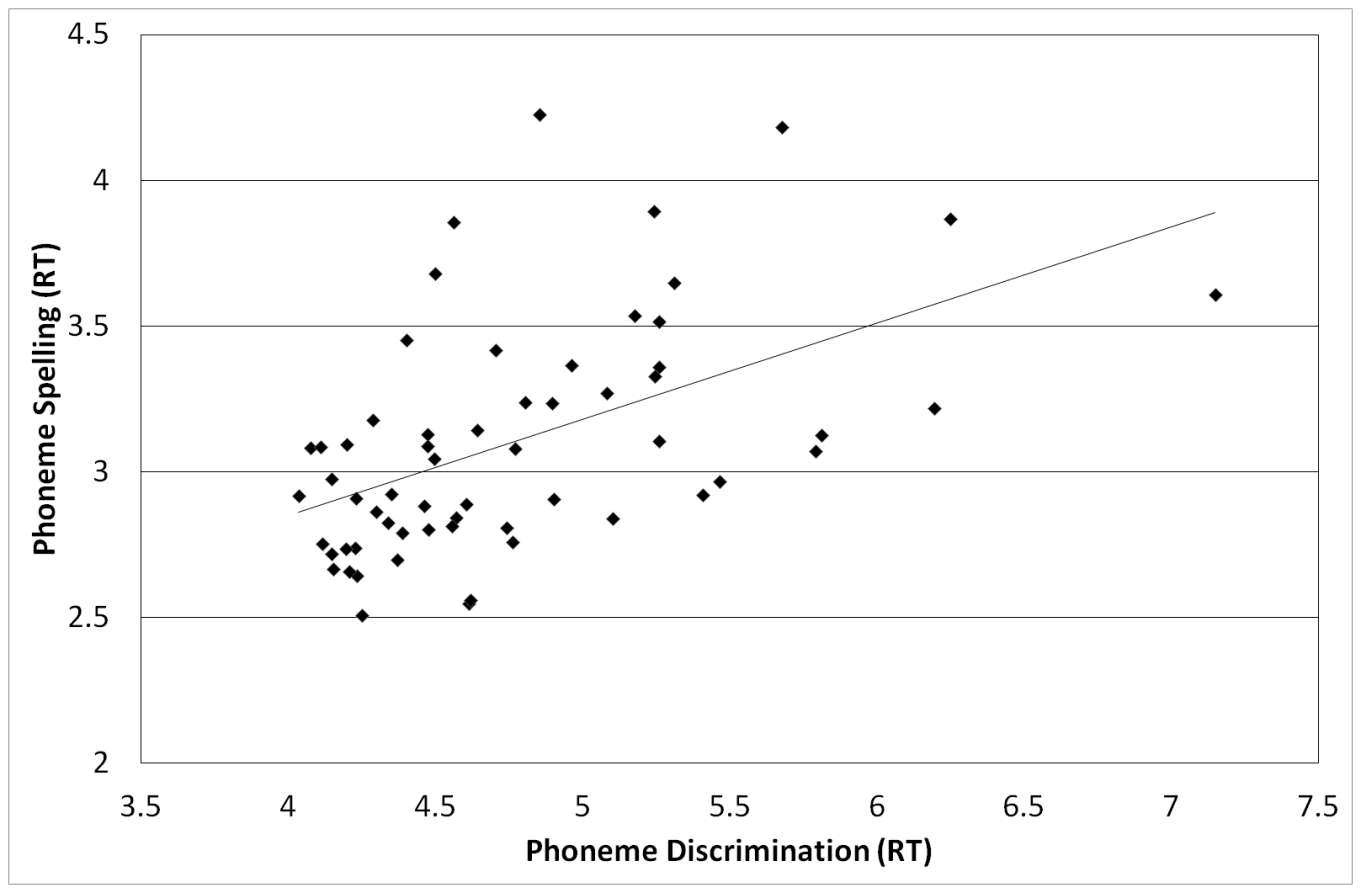
Correlation between phoneme discrimination and phoneme spelling, *r*(59) =  .52, *p* < .001.

None of the auditory processing tasks correlated with one another when analyzed in terms of accuracy. When analyzed in terms of (RT), all were moderately to strongly correlated. Nonlinguistic auditory processing was significantly correlated with both the phoneme discrimination measure (*r*(59)  =  .60, *p*< .001) and the phoneme spelling measure (*r*(58) =  .45, *p*< .001). The two linguistic auditory processing measures correlated significantly with one another, *r*(59) =  .52, *p*< .001. In short, all of the measures of auditory processing correlated with one another in terms of speed of processing, but not in terms of accuracy of responses (see Figures 1–3 for scatterplots of reaction times). No corrections were used for multiple tests.

### Linguistic Auditory Processing and Spelling

Correlational analyses were conducted both for the group as a whole, and for phonological and nonphonological spellers separately. We did not find any significant correlations when assessing accuracy of responses, but found more interesting results when considering RT. Looking at the group as a whole, RT for the phoneme discrimination task did not correlate significantly with spelling ability, *r*(60) =  .098, *p* =  .447. RT for the phoneme spelling task did not correlate significantly with spelling, *r*(59) = −.187, *p* =  .149.

**Figure 4:**
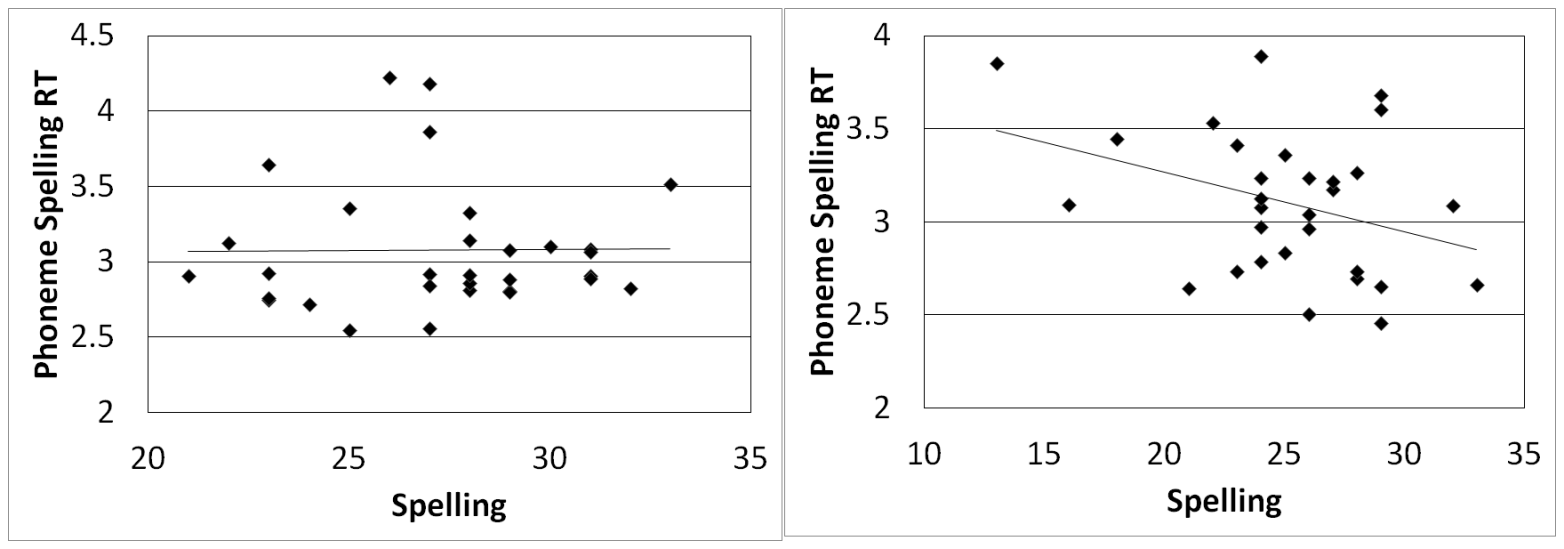
Correlation between phoneme spelling RT and spelling ability among nonphonological spellers, *r*(29) =  .009, *p* =  .960 (left), compared with correlation between phoneme spelling RT and spelling ability among phonological spellers, *r*(28) = −.353, *p* =  .056 two-tailed and .028 one-tailed (right).

When we divided the participants by spelling strategy, a slightly different picture emerged. RTs for the phoneme discrimination task did not correlate significantly with spelling ability for nonphonological spellers (*r*(30) = −.06, *p* =  .728) or for phonological spellers (*r*(28) =  .184, *p* =  .330). RTs for the phoneme spelling task did not correlate significantly with spelling ability for nonphonological spellers, *r*(29) =  .009, *p* =  .960 (see Figure 4). However, the correlation between the phoneme spelling task spelling ability for phonological spellers was significant, *r*(28) = −.353, *p* =  .028 (1-tailed) (See Figure 4). So, among phonological spellers only, there was a slight tendency for those who were quicker in the phoneme spelling task to be better spellers. No corrections were used for multiple tests.

A regression analysis was conducted to shed more light on these correlations. As predicted, the nonphonological spellers did not demonstrate a relationship between auditory processing and spelling (F(6,23) =  .378, *p* = .885). Phonological spellers showed several notable trends in our analysis. The RT for both the phoneme discrimination and for the phoneme spelling approached significance (*b* =  .586, *t*(30) = 1.720, *p* =  .100 and *b* = −.446, *t*(30) = −1.987, *p* =  .060, respectively). Thirty-seven percent of the variability in spelling ability was accounted for by the auditory processing factors combined (R^2^ =  .372).

### Nonlinguistic Auditory Processing and Spelling

For the group as a whole, accuracy on the nonlinguistic auditory processing task did not significantly correlate with spelling, *r*(60) =  .165, *p* =  .200.

**Figure 5:**
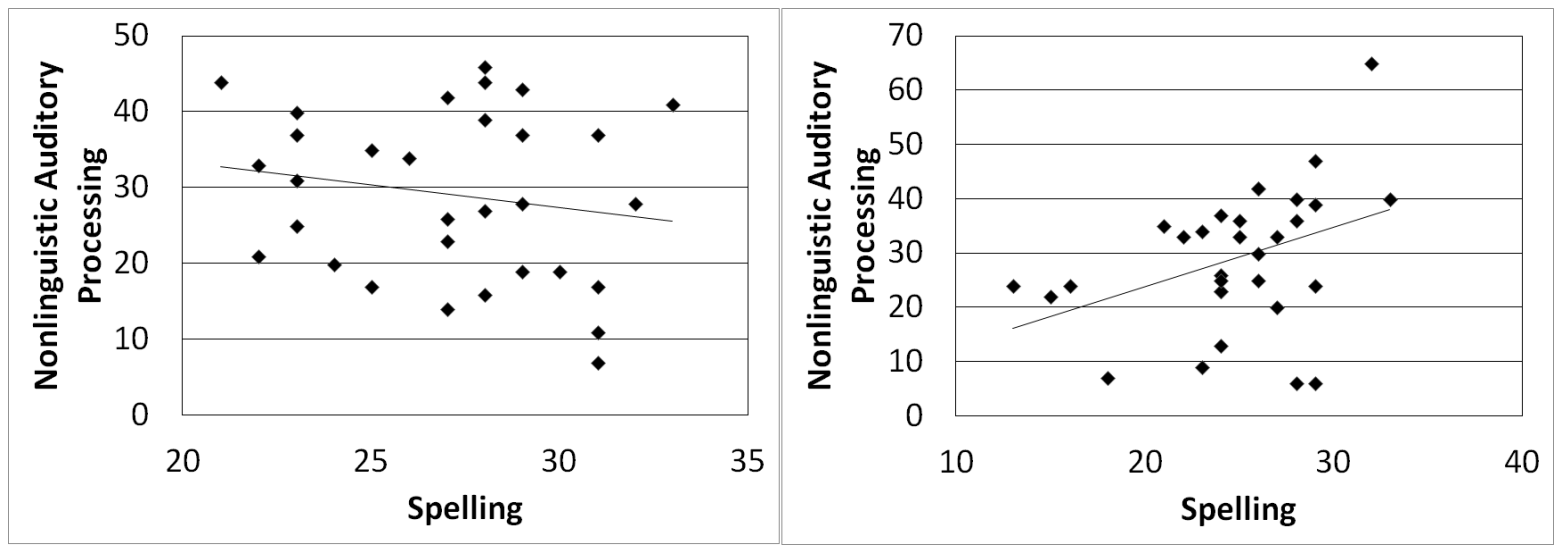
Correlation between nonlinguistic auditory processing accuracy and spelling ability among nonphonological spellers, *r*(29) = −.179, *p* =  .335 (left), compared with correlation between nonlinguistic auditory processing accuracy and spelling ability among phonological spellers, *r*(29) =  .385, *p* =  .032 (right).

Accuracy on the nonlinguistic auditory processing task did not correlate significantly with spelling for nonphonological spellers, *r*(29) = −.179, *p* =  .335 (See Figure 5). Accuracy on the nonlinguistic auditory processing task did, however, correlate significantly with spelling for phonological spellers, *r*(29) =  .385, *p* =  .032 (See Figure 5). Among phonological spellers only, there was a tendency for those who performed better on the nonlinguistic auditory processing task to be better spellers. No corrections were used for multiple tests.

## Discussion

The present study combines areas of research linking linguistic auditory processing to spelling, e.g., [33], conflicting research investigating links between nonlinguistic auditory processing to spelling (e.g., [34] v. [28]), and studies suggesting that strategy choice may impact these links, e.g., [12]. The current effort represents the first study in which both auditory processing and strategy choice are considered, and performance on both is compared. This unique approach adds to the literature and serves as a foundation for research in these areas in the future. Based on the analyses, in sum, the answers to our research questions are: a) the different types of auditory processing appear to be somewhat related to one another, b) both linguistic and nonlinguistic processing appear related to spelling, and c) the relationship between auditory processing and spelling appears to hold for individuals who rely heavily on phonological strategies but not for those who do not.

### Linguistic Versus Nonlinguistic Auditory Processing

Although the results from this study do not provide definitive proof that nonlinguistic auditory processing underlies linguistic auditory process, the results of this study do indicate a possible relationship between linguistic and nonlinguistic auditory processing. This finding provides potential support for the idea of lower-level auditory skills being at the root of the phonological processing problems often reported in those with low literacy skills. It may not be possible to tease linguistic and nonlinguistic auditory processing apart to a sufficient extent to determine that one is the cause of the other. However, at the very least, this result suggests that linguistic and nonlinguistic auditory processing are connected, and that both are important for spelling. These findings could have implications for screening for future spelling ability and also for identifying different developmental ability and disabilities. That is, if there is a sufficiently strong overlap between linguistic and nonlinguistic auditory processing, it may not be necessary to test both when screening.

We found correlations for RTs only, rather than for accuracy scores; this result could have at least two potential explanations. One possibility is that there may have been ceiling and floor effects (scores were, in general, quite high on the linguistic tasks and fairly low on the nonlinguistic tasks), thus making RT the only indicator with enough variability to show results. Another possibility is that individual differences in participants' general speed of processing may have contributed significantly to this result. Thus, this particular result should be regarded with caution.

### Linguistic Auditory Processing and Spelling

Analyses yielded a moderate correlation between linguistic auditory processing and spelling for phonological spellers. On the phoneme spelling task, this correlation was for RT only. While the lack of a significant correlation for accuracy on the phoneme spelling task may have been due to the ceiling effects seen on this test, the significant correlation with RT on this same task is still informative. This finding suggests that rapid linguistic auditory processing is important for efficient use of phonological strategies, even for adults who are experienced spellers. It is therefore possible that more challenging linguistic auditory processing tasks would produce different results. In accordance with the correlational findings, our regression model also indicated that a marked proportion of the variance in spelling ability among phonological spellers was accounted for by auditory processing.

While significant in terms of the correlations, and approaching significance in terms of the regression analysis, note that the relationship between linguistic auditory processing and spelling is not as strong as those reported in past studies, for instance [7]–[8]. Of note, these studies focused on differences between participants with and without reading disabilities, and although further research would be necessary to draw any definitive conclusions, a probable explanation is that auditory processing plays a smaller role in influencing individual differences within a nondisabled population than it does in the differences between groups that do or do not have reading disabilities.

### Nonlinguistic Auditory Processing and Spelling

A significant correlation between frequency discrimination and spelling ability for phonological spellers was identified. This finding suggests that some of the conflicts in pre-existing literature may have their roots in different strategy choices among participants. Some researchers, e.g., [36] have reported auditory processing deficits in only sub-groups of individuals with literacy impairments; this research may reflect sub-groups in terms of strategy choice.

### Use of Phonology

The significant negative correlation between use of phonology and spelling ability was not surprising. Heavy reliance on phonological strategies into adulthood has been associated with poor spelling and poor reading, e.g., [4], [13]–[14], [18]. Ideally, spelling ability would be controlled for between groups of phonological and nonphonological spellers; however, among adults, phonological spellers are usually less-accurate spellers. Note that this is not a result we would necessarily expect to replicate among younger spellers.

## Conclusions

The main finding of this study is that there appears to be a link between both linguistic and nonlinguistic auditory processing and spelling ability. This link is not as strong as has been reported by researchers who have compared people with disabilities to those whose reading and spelling is typically developing, but it is nevertheless significant in nondisabled spellers. The correlation between auditory processing and spelling ability existed only in the phonological spelling group. Thus, strategy choice appears to be a significant mediating variable, worthy of further investigation. Related to this is the implication that a spelling strategy that is adaptive for one person may not be adaptive for another. Phonological strategies are clearly less adaptive here for individuals with less accurate auditory processing than for those with more accurate auditory processing.

## Future Directions

Research to date has focused on improving reading and/or spelling in individuals who are experiencing difficulty. It may be possible, however, to identify individuals before they begin to experience problems. For this reason, replication of this study with children is currently underway. A study is also currently being devised with the intention of following children longitudinally in order to assess spelling achievement in children who have been screened using these auditory processing tasks. The goal would be to determine the usefulness of these auditory processing tasks as screening tools as well as to identify meaningful cutoffs for assessment.

Remediation is another avenue for consideration. If auditory processing is related to spelling for phonological spellers only, it is possible that changing strategies would be useful for people experiencing difficulty with auditory processing. Should individuals who have difficulties with auditory processing be provided with remedial phonics instruction, as they often are? Or should these same individuals receive instruction in alternate spelling strategies? Would individuals benefit from both types of instruction? An in-depth longitudinal study comparing methods of remediation is needed to address and improve methods for helping individuals who are experiencing difficulties with spelling.

## Supporting Information

Appendix S1 **Tone pairs used in nonlinguistic auditory processing task.**
(DOCX)

Appendix S2 **Phoneme pairs used in phoneme matching task.**
(DOCX)

Appendix S3 **Phonemes and spelling choices used in phoneme spelling task.**
(DOCX)
