# Supplementary material for: Strategy Choice Mediates the Link between Auditory Processing and Spelling
Source: PLoS One. 2014 Sep 8;9(9):e107131. doi: 10.1371/journal.pone.0107131 (PMC4157809; doi:10.1371/journal.pone.0107131)
Supplement: Appendix S1 — Tone pairs used in nonlinguistic auditory processing task. (DOCX) [file pone.0107131.s001.docx]

Appendix A

Tone Pairs

*1000Hz Block*

1000Hz-1000Hz X 2

1000Hz-1002Hz 1000Hz-998Hz

1000Hz-1004Hz 1000Hz-996Hz

1000Hz-1006Hz 1000Hz-994Hz

1000Hz-1008Hz 1000Hz-992Hz

1000Hz-1010Hz 1000Hz-990Hz

1000Hz-1012Hz 1000Hz-988Hz

1000Hz-1014Hz 1000Hz-986Hz

1000Hz-1016Hz 1000Hz-984Hz

1000Hz-1018Hz 1000Hz-982Hz

1000Hz-1020Hz 1000Hz-980Hz

*1500Hz Block*

1500Hz-1500Hz X 2

1500Hz-1502Hz 1500Hz-1498Hz

1500Hz-1504Hz 1500Hz-1496Hz

1500Hz-1506Hz 1500Hz-1494Hz

1500Hz-1508Hz 1500Hz-1492Hz

1500Hz-1510Hz 1500Hz-1490Hz

1500Hz-1512Hz 1500Hz-1488Hz

1500Hz-1514Hz 1500Hz-1486Hz

1500Hz-1516Hz 1500Hz-1484Hz

1500Hz-1518Hz 1500Hz-1482Hz

1500Hz-1520Hz 1500Hz-1480Hz

*2000Hz Block*

2000Hz-2000Hz X 2

2000Hz-2002Hz 2000Hz-1998Hz

2000Hz-2004Hz 2000Hz-1996Hz

2000Hz-2006Hz 2000Hz-1994Hz

2000Hz-2008Hz 2000Hz-1992Hz

2000Hz-2010Hz 2000Hz-1990Hz

2000Hz-2012Hz 2000Hz-1988Hz

2000Hz-2014Hz 2000Hz-1986Hz

2000Hz-2016Hz 2000Hz-1984Hz

2000Hz-2018Hz 2000Hz-1982Hz

2000Hz-2020Hz 2000Hz-1980Hz
