# Supplementary material for: Strategy Choice Mediates the Link between Auditory Processing and Spelling
Source: PLoS One. 2014 Sep 8;9(9):e107131. doi: 10.1371/journal.pone.0107131 (PMC4157809; doi:10.1371/journal.pone.0107131)
Supplement: Appendix S2 — Phoneme pairs used in phoneme matching task. (DOCX) [file pone.0107131.s002.docx]

Appendix B

Phoneme Pairs

(Each given as spelling/phonetic symbol)

*Matching Confusable Dissimilar*

k/k-k/k k/k-g/g k/k-s/s

g/g-g/g f/f-th/θ g/g-f/f

b/b-b/b b/b-d/d b/b-ch/t∫

d/d-d/d d/d-t/t d/d-sh/∫

z/z-z/z z/z-s/s z/z-th/θ

s/s-s/s j/dƷ-ch/t∫ s/s-f/f

v/v-v/v v/v-f/f v/v-d/d

f/f-f/f v/v-th/ ð f/f-t/t

ch/t∫- ch/t∫ /ch/t∫-sh/∫ ch/t∫- p/p

sh/∫- sh/∫ p/p-b/b sh/∫-b/b

th/θ- th/θ d/d-j/ dƷ v/v-k/k

t/t-t/t t/t-ch/ t∫

j/ dƷ- j/ dƷ j/ dƷ-v/v

p/p-p/p ch/ t∫- th/θ

th/ ð- th/ ð p/p-z/z

b/b- sh/∫

th/ ð-g/g
