# Supplementary material for: Strategy Choice Mediates the Link between Auditory Processing and Spelling
Source: PLoS One. 2014 Sep 8;9(9):e107131. doi: 10.1371/journal.pone.0107131 (PMC4157809; doi:10.1371/journal.pone.0107131)
Supplement: Appendix S3 — Phonemes and spelling choices used in phoneme spelling task. (DOCX) [file pone.0107131.s003.docx]

Appendix C

Phoneme Spelling Task Sounds and Response Options

**Sound:** k/k **Sound**: f/f

**Options:** k g s **Options**: f th t

**Sound:** g/g **Sound**: th/θ

**Options**: g k f **Options**: th f d

**Sound**: b/b **Sound**: d/d

**Options**: b d ch **Options**: d t j

**Sound**: d/d **Sound**: t/t

**Options**: d b sh **Options**: t d ch

**Sound**: z/z **Sound**: j/dƷ

**Options**: z s th **Options**: j ch v

**Sound**: s/s **Sound**: ch/t∫

**Options**: s z f **Options**: ch j th

**Sound**: v/v **Sound**: p/p

**Options**: v f d **Options**: p b z

**Sound**: f/f **Sound**: b/b

**Options**: v f t **Options**: b p sh

**Sound**: ch/t∫ **Sound**: v/v

**Options**: ch sh p **Options**: v th k

**Sound**: sh/∫ **Sound**: th/ð

**Options**: sh ch b **Options**: th v g
